# Supplementary material for: Synthetic images aid the recognition of human-made art forgeries
Source: PLoS One. 2024 Feb 14;19(2):e0295967. doi: 10.1371/journal.pone.0295967 (PMC10866502; doi:10.1371/journal.pone.0295967)
Supplement: S1 Appendix — 1. Image generation using StyleGAN. This section presents details regarding the training configurations employed by StyleGAN3. Additionally, we list the numbers of images used for training, along with the corresponding quality of the generated results, sorted by categories. Furthermore, we include visual representations of sample images generated after the training. 2. Classification results for Modigliani and Raphael. The entire workflow presented for the artist Vincent van Gogh in the main manuscript has also been performed for Amedeo Modigliani and Raphael (Raffaello Sanzio da Urbino). The results can be found in this section. (PDF) [file pone.0295967.s001.pdf]

Supplemental Material: S1 Appendix

Johann Ostmeyer[1¶], Ludovica Schaerf[2¶], Pavel Buividovich[1*], Tessa Charles[3], Eric Postma[4], Carina Popovici[2]

1 Department of Mathematical Sciences, University of Liverpool, Liverpool L69 3BX, United Kingdom
2 Art Recognition AG, Soodmattenstrasse 4, CH-8134 Adliswil, Switzerland
3 Department of Physics, University of Liverpool, Liverpool L69 3BX, United Kingdom
4 Cognitive Science & AI, Tilburg University, 5037 AB Tilburg, the Netherlands

¶These authors contributed equally to this work.
* Pavel.Buividovich@liverpool.ac.uk

1 Evaluation of the quality of synthetic images generated by StyleGAN

In this section of the supplementary information to the manuscript "Synthetic images aid the recognition of human-made art forgeries" we detail the procedure employed for the training of StyleGAN3 [1], the generation of images as well as their quality.

The training was performed independently on genre-based subsets of Wikiart (www.wikiart.org), with the number of images of each subset and the corresponding genre listed in Tab. 2. We trained each genre starting from white noise (i.e. no pre-training) with a resolution of 256×256 pixels. The portraits analysed in the main manuscript were trained in an independent additional run using a higher resolution of 512×512. The corresponding hyperparameters we used for the training with StyleGAN3 are listed in Tab. 1.

Table 1. Hyperparameters used for the training with StyleGAN3 [1].
The full command run for training would read python train.py -cfg=stylegan2 -kimag=5000 <hyperparameters>. We also used -snap=50 -tick=1 -metrics==kid50k_full,fid50k_full to monitor the progress.

resolution	-batch=	-gamma=	-cbase=	-glr=	-dlr=	-mbstd-group=
256×256	16	1	16384	0.001	0.001	4
512×512	12	5		0.001	0.001	3

It is interesting to note that the latest alias-free version included in StyleGAN3 turned out to perform worse on artworks than the older version StyleGAN2 [2], in our case realized by the corresponding flag natively provided in StyleGAN3. This is likely a consequence of local hard transitions, often featured by brush strokes, which tend to be smeared out by the translationally invariant StyleGAN3.

Tab. 2 provides an overview over the number of images used for training and the resulting image qualities achieved by the GAN for different image types with a resolution of 256×256. The Fréchet Inception Distance (FID) is the state of the art estimator for image quality that is closest to human perception as a rule. A high FID (ca. 20 or more) usually signifies bad results, while a low FID (here less than 20) indicates that the images are reasonably realistic. However, this rule has notable exceptions, in our case 'history and genre paintings' as well as 'still and flower

Table 2. Training data sets.
Number of images with resolution at least 256×256, and quality of the training results using `StyleGAN3`. We provide the Fréchet Inception Distance (FID) as a metric for the quality of the generated images (lower is better).

Type	No. images	FID
portraits and self-portraits	10 380	13.6
abstract	2571	42.8
animals	863	93.7
cityscapes	3211	18.5
figurative and allegorical	2694	40.4
history and genre paintings	10 557	14.0
illustrations and sketches	880	78.5
landscapes	10 795	10.2
nude paintings	1147	63.0
religious paintings	4663	21.0
still and flower paintings	2729	39.4

paintings'. Most humans would readily agree that the former category produced unsatisfactory results (see fig. 1, top left) while the latter succeeded with the flowers at least (see fig. 1, bottom right), both contrary to the FID predictions.

There is an overall trend that a larger training set results in higher quality images as could have been expected. However, even with similar sample sizes, some categories fare much better than others. Some examples are shown in Fig. 1 where the images in the top row ('history and genre paintings' and 'landscapes') have training data sets of similar size. The data sets 'figurative and allegorical' and 'still and flower paintings' are also similar in size. Their representatives in the bottom row have extremely different quality as well.

We speculate on several causes which can, at least partially, be responsible for such differences in generative quality. One possibility and known issue of GANs is a poor convergence of the optimizer during the training process. A high number of minuscule details is certainly prohibitive when learning on such low resolutions, for instance, many people in the historical paintings, each with facial features. Both, the image resolution and the network capacities are insufficient to resolve this kind of details. In addition, a large diversity of images poses a difficulty because the GAN might not be able to identify reoccurring features and is incapable of generalizations. This is most likely the pivotal problem with the figurative and allegorical paintings.

A separate training run has been performed on images with the higher resolution of 512×512 for the 'portraits and self-portraits' category, which serves as a base for all the analysis in the main Manuscript. These GAN images are used for the benchmarks below. These are trained on a smaller training set (only 7983 of the 10 380 images had a sufficient resolution) and a higher number of network parameters (59 259 432 instead of 48 768 547 parameters in total).

Figure 1. Representative 256×256 images.
Categories used are ‘history and genre paintings’ (top left), ‘landscapes’ (top right), ‘figurative and allegorical’ (bottom left), and ‘still and flower paintings’ (bottom right).

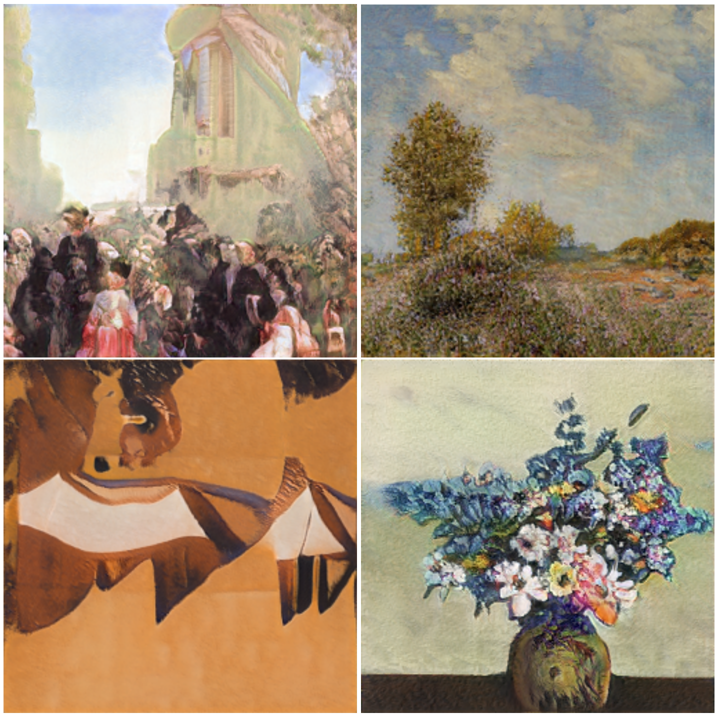

2 Classification results for Modigliani and Raphael

This section of the supplementary information to the manuscript “Synthetic images aid the recognition of human-made art forgeries” complements the results presented in the manuscript for the artist Vincent van Gogh with analogous studies using portraits by Amedeo Modigliani and Raphael (Raffaello Sanzio da Urbino). The entire procedure (from data generation to classification and analysis) is identical with that employed for van Gogh and we refer to the main manuscript for the details.

Data sets

The compositions of the training datasets are listed in Tabs. 3 and 4 with representative images of each category displayed in Figs. 2 and 3 for Modigliani and Raphael, respectively.

Modigliani’s dataset contains 100 original artworks, 58 ’proxies’, 21 ’imitations’, and the same amount of synthetic fakes as van Gogh, 30 per type. Raphael, on the other side, contains 206 original artworks, 96 ’proxies’, and 99 ’imitations’, also with the same number of synthetic images.

All displayed images of human-made paintings [3–6] are in the public domain.

Table 3. Composition of the Modigliani dataset.

Image set	Number of images	Patches
authentic	100	1812
imitations	21	269
proxies	58	1160
tuned GANs	30	150
raw GANs	30	150
diffusion	30	150

Table 4. Composition of the Raphael dataset.

Image set	Number of images	Patches
authentic	206	2756
imitations	99	1293
proxies	96	1296
tuned GANs	30	150
raw GANs	30	150
diffusion	30	150

Figure 2. Illustration of human-made (top row) and synthetic (bottom row) Modigliani images.
"Nu couché" (Reclining nude) by Amedeo Modigliani [3] (square cropped, left), imitation of Modigliani's "Portrait de Chaïm Soutine" [4, 7] (square cropped, top), fine-tuned GAN generated image in style of Modigliani (bottom left), and Stable Diffusion generated image in style of Modigliani (bottom right).

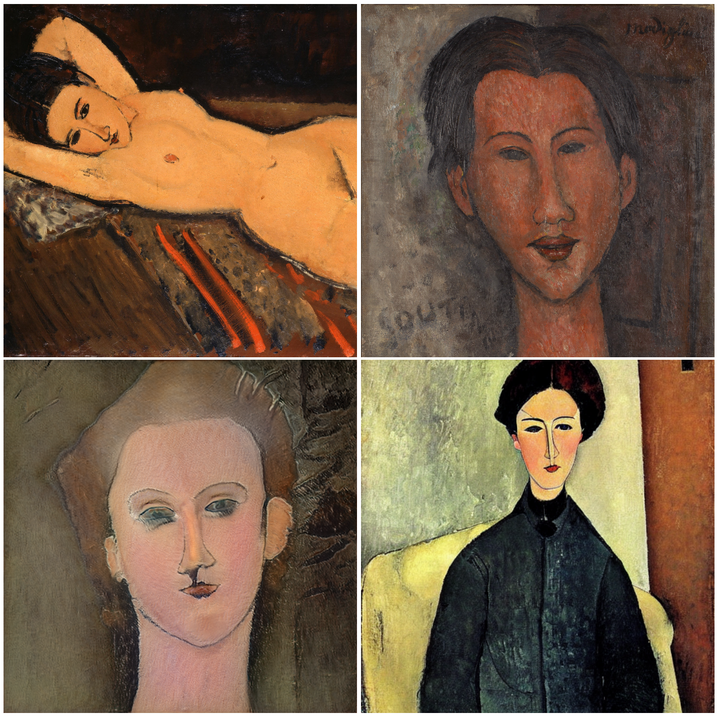

Figure 3. Illustration of real (top row) and synthetic (bottom row) Raphael images.
"Madonna with Child" by Raffaello Sanzio [5] (square-cropped, top left), "Portrait of a Young Man in Red" by the Circle of Raphael [6] (top right), fine-tuned GAN generated image in the style of Raffaello (bottom left), and Stable Diffusion generated image in the style of Raffaello (bottom right).

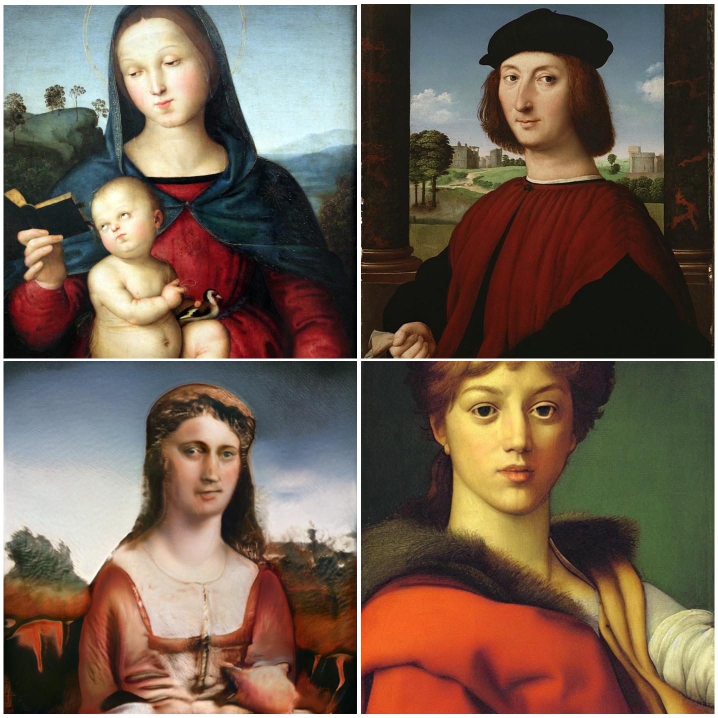

Detection of human forgeries

In the following we report the results of the classification experiments: Modigliani with human-made forgeries in the training set (Tab. 5, top panels of Fig. 4), without forgeries (Tab. 6, third row of Fig. 4), Raphael with forgeries (Tab. 7, second row of Fig. 4), without forgeries (Tab. 8, bottom panels of Fig. 4).

The accuracy of the classification of original paintings is consistently high and fully compatible with the results obtained for van Gogh paintings in the main manuscript. The accuracy of forgery classification on the other hand is lower than that observed for van Gogh in most cases, especially when no human-made forgeries are included in the training set. It is important to note that this is in no way contradicting the conclusions drawn in the main manuscript since, regardless of absolute numbers, these accuracies improve significantly in all considered cases when synthetic forgeries are added to the training data.

Table 5. Performance for Modigliani on different tests after training with forgeries.

training contrast set	model architecture	accuracy forgeries	accuracy originals
no synthetic	Swin Base	0.580(101)	0.932(19)
no synthetic	EfficientNet B0	0.461(97)	0.881(9)
raw GANs	Swin Base	0.561(137)	0.920(15)
raw GANs	EfficientNet B0	0.531(118)	0.869(13)
tuned GANs	Swin Base	0.491(138)	0.926(14)
tuned GANs	EfficientNet B0	0.516(121)	0.835(15)
diffusion	Swin Base	0.553(117)	0.932(15)
diffusion	EfficientNet B0	0.484(83)	0.865(11)
diffusion+GANs	Swin Base	0.605(100)	0.917(17)
diffusion+GANs	EfficientNet B0	0.543(146)	0.843(20)

Table 6. Performance for Modigliani on different tests after training without forgeries.

training contrast set	model architecture	accuracy forgeries	accuracy originals
no synthetic	Swin Base	0.115(51)	0.998(1)
no synthetic	EfficientNet B0	0.263(58)	0.971(4)
raw GANs	Swin Base	0.153(53)	0.997(1)
raw GANs	EfficientNet B0	0.293(60)	0.961(6)
tuned GANs	Swin Base	0.193(62)	0.991(2)
tuned GANs	EfficientNet B0	0.337(74)	0.955(7)
diffusion	Swin Base	0.221(49)	0.992(3)
diffusion	EfficientNet B0	0.326(65)	0.975(5)
diffusion+GANs	Swin Base	0.262(57)	0.983(5)
diffusion+GANs	EfficientNet B0	0.422(87)	0.944(7)

Table 7. Performance for Raphael on different tests after training with forgeries.

training contrast set	model architecture	accuracy forgeries	accuracy originals
no synthetic	Swin Base	0.926(26)	0.804(22)
no synthetic	EfficientNet B0	0.681(35)	0.713(16)
raw GANs	Swin Base	0.902(22)	0.835(21)
raw GANs	EfficientNet B0	0.736(33)	0.718(15)
tuned GANs	Swin Base	0.906(27)	0.820(20)
tuned GANs	EfficientNet B0	0.746(30)	0.704(18)
diffusion	Swin Base	0.899(22)	0.838(17)
diffusion	EfficientNet B0	0.701(37)	0.707(20)
diffusion+GANs	Swin Base	0.924(31)	0.797(20)
diffusion+GANs	EfficientNet B0	0.719(27)	0.693(14)

Table 8. Performance for Raphael on different tests after training without forgeries.

training contrast set	model architecture	accuracy forgeries	accuracy originals
no synthetic	Swin Base	0.009(2)	0.962(9)
no synthetic	EfficientNet B0	0.083(10)	0.887(11)
raw GANs	Swin Base	0.071(11)	0.940(9)
raw GANs	EfficientNet B0	0.127(11)	0.851(15)
tuned GANs	Swin Base	0.039(8)	0.950(8)
tuned GANs	EfficientNet B0	0.111(9)	0.877(12)
diffusion	Swin Base	0.066(12)	0.953(8)
diffusion	EfficientNet B0	0.127(12)	0.848(12)
diffusion+GANs	Swin Base	0.087(13)	0.950(10)
diffusion+GANs	EfficientNet B0	0.155(16)	0.829(14)

Figure 4. Accuracies of different models.
Classification results for Modigliani and Raphael based on tables 5 to 8. The horizontal dotted line shows the baseline without synthetic images in the training data.

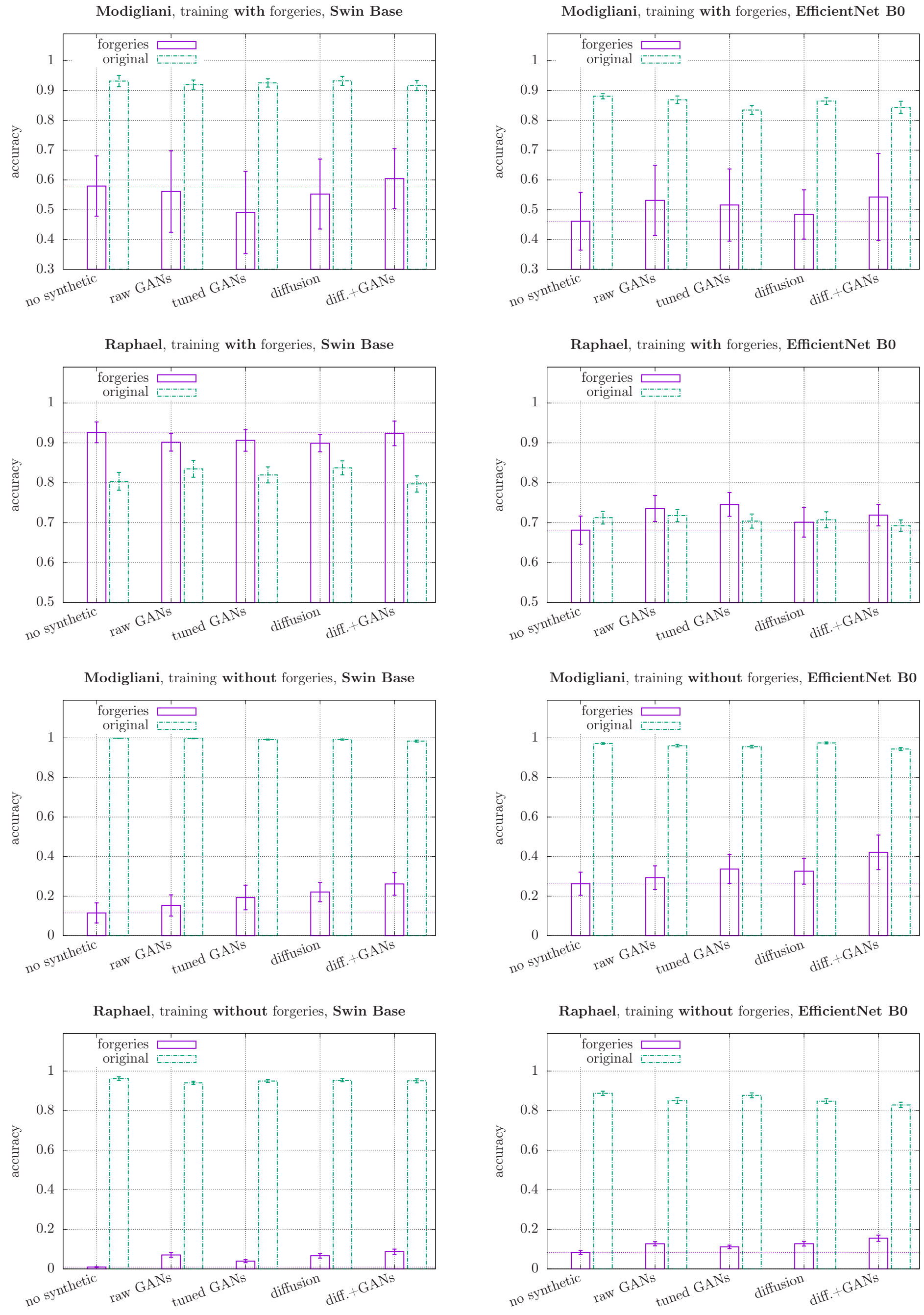

Detection of synthetic images

As in the main manuscript, we perform a sanity check benchmarking our classifiers on synthetic forgery detections. The results can be found in Tabs. 9 and 10 as well as Fig. 5. They consistently support the conclusions that high detection accuracies for a given generator architecture can be obtained when data from this generator is included in the training set and that the detection of adversarial attacks is not reliable when this particular generator is not used for training.

Table 9. Accuracy of synthetic forgery detection for Modigliani.

training contrast set	model architecture	accuracy Stable Diffusion	accuracy tuned GANs
no synthetic	Swin Base	0.656(93)	0.027(8)
no synthetic	EfficientNet B0	0.444(46)	0.071(13)
raw GANs	Swin Base	0.790(58)	0.327(47)
raw GANs	EfficientNet B0	0.495(68)	0.177(32)
tuned GANs	Swin Base	0.792(51)	0.809(46)
tuned GANs	EfficientNet B0	0.590(59)	0.633(45)
diffusion	Swin Base	0.943(20)	0.045(10)
diffusion	EfficientNet B0	0.832(37)	0.084(19)
diffusion+GANs	Swin Base	0.980(9)	0.934(21)
diffusion+GANs	EfficientNet B0	0.892(31)	0.666(34)

Table 10. Accuracy of synthetic forgery detection for Raphael.

training contrast set	model architecture	accuracy Stable Diffusion	accuracy tuned GANs
no synthetic	Swin Base	0.778(59)	0.322(29)
no synthetic	EfficientNet B0	0.215(36)	0.164(19)
raw GANs	Swin Base	0.781(84)	0.643(32)
raw GANs	EfficientNet B0	0.216(34)	0.279(16)
tuned GANs	Swin Base	0.841(46)	0.932(10)
tuned GANs	EfficientNet B0	0.276(32)	0.599(11)
diffusion	Swin Base	0.955(24)	0.387(45)
diffusion	EfficientNet B0	0.539(33)	0.225(15)
diffusion+GANs	Swin Base	0.971(9)	0.957(30)
diffusion+GANs	EfficientNet B0	0.544(33)	0.605(23)

Figure 5. Accuracies of different models for synthetic data.
Based on the results shown in tables 9 and 10.

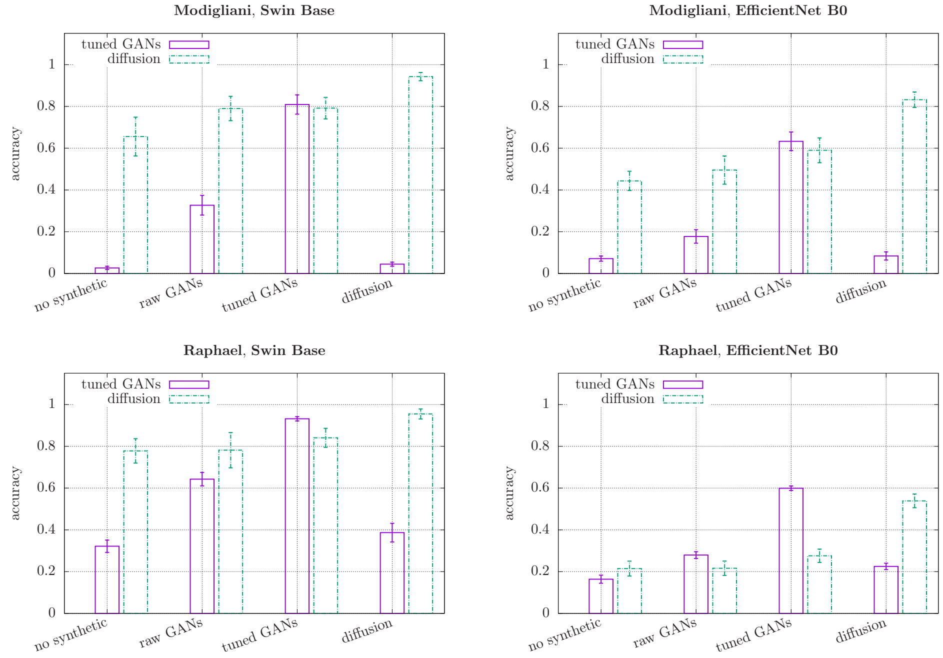

References

1. Karras T, Aittala M, Laine S, Härkönen E, Hellsten J, Lehtinen J, et al.. Alias-Free Generative Adversarial Networks; 2021. Advances in Neural Information Processing Systems 34 (NeurIPS 2021). Available from: `https://arxiv.org/abs/2106.12423`.

2. Karras T, Laine S, Aittala M, Hellsten J, Lehtinen J, Aila T. Analyzing and Improving the Image Quality of StyleGAN. In: 2020 IEEE/CVF Conference on Computer Vision and Pattern Recognition (CVPR); 2020. p. 8107–8116. Available from: `https://dx.doi.org/10.1109/CVPR42600.2020.00813`.

3. Modigliani A. Nu couché; 1916. Available from: `https://commons.wikimedia.org/wiki/File:Amedeo_Modigliani,_1916,_Reclining_Nude_(Nu_couch%C3%A9),_oil_on_canvas,_65.5_x_87_cm,_Foundation_E.G._B%C3%BChrle.jpg`.

4. Modigliani A. Portrait de Chaïm Soutine réalisé en 1917; 1917. Available from: `https://commons.wikimedia.org/wiki/File:Amedeo_Modigliani_-_Cha%C3%AFm_Soutine_-_1917_-_001.jpg`.

5. Raffaello Sanzio da Urbino. Madonna Solly; 1502. Available from: `https://commons.wikimedia.org/wiki/File:1502_Raffael_Madonna_Solly_anagoria.JPG`.

6. Circle of Raphael. Portrait of a Young Man in Red; about 1505. Available from: `https://www.getty.edu/art/collection/object/103REA`.

7. Cascone S. Visitors to Major Modigliani Exhibition Demand a Refund After an Expert Concludes the Majority of Works Were Fake; 2018. Available from:

https://news.artnet.com/art-world/
art-lovers-demand-refund-fake-modigilani-exhibition-1197245.
